# A follow-up on the hesperetin issue in modelling the first electrochemical oxidation potential and antioxidant activity of flavonoids

**DOI:** 10.2478/aiht-2024-75-3823

**Published:** 2024-03-29

**Authors:** Ante Miličević

**Affiliations:** Institute for Medical Research and Occupational Health, Zagreb, Croatia

Dear Editor,

In Volume 70 (pages 134–139) of *Arhiv za higijenu rada i toksikologiju* – *Archives of Industrial Hygiene and Toxicology*, I published a paper entitled “The relationship between antioxidant activity, first electrochemical oxidation potential, and spin population of flavonoid radicals” (1). The paper detected a problem with hesperetin, a flavonoid (flavanone) with 4′-methoxy and 3′-hydroxyl groups on the B ring. That problem was later resolved in a paper published in the *Journal of Molecular Liquids* (2021;335:116223) on a set of 29 flavonoids (2), which I believe is worth reporting as a follow-up to my aforementioned article published in the *Archives*.

**Figure 1:**
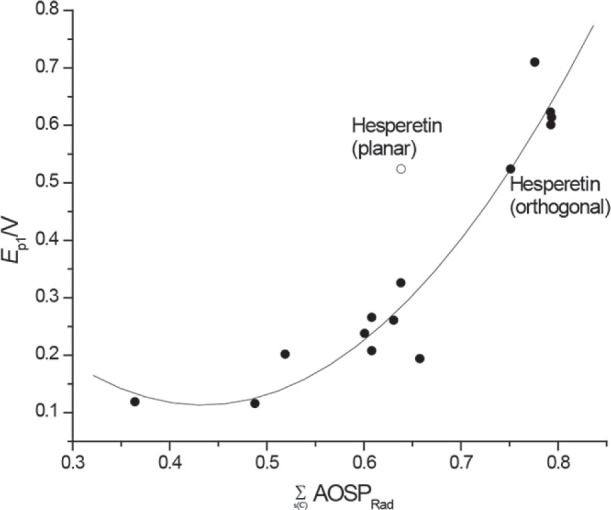
The dependence of experimental *E*_p1_ (pH = 7) on $\sum_{\text{s}(\text{C})}{\text{AOSP}}_{\text{Rad}}$ , calculated using the PM6 method, for 14 flavonoids from (1). Empty circle represents $\sum_{\text{s}(\text{C})}{\text{AOSP}}_{\text{Rad}}$ of hesperetin calculated using methoxy group planar with the B ring plane [as in (1)]. When the methoxy group was set orthogonally to the B ring plane (filled circle), the $\sum_{\text{s}(\text{C})}{\text{AOSP}}_{\text{Rad}}$ of hesperetin fit the regression model, yielding *R*^2^=0.930, SE=0.053, and SE_cv_=0.069

**Figure 2:**
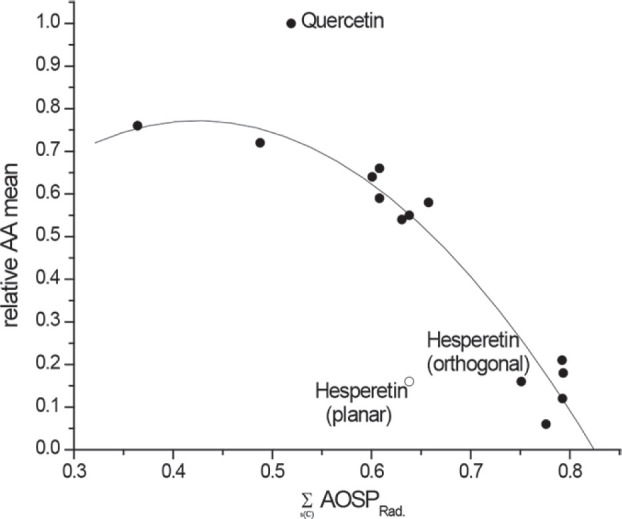
The dependence of experimental relative AA mean on $\sum_{\text{s}(\text{C})}{\text{AOSP}}_{\text{Rad}}$ , calculated using the PM6 method for the set of 14 flavonoids from (1). Empty circle represents $\sum_{\text{s}(\text{C})}{\text{AOSP}}_{\text{Rad}}$ of hesperetin calculated using methoxy group planar with the B ring plane [as in (1)]. When the methoxy group was set orthogonally to the B ring plane (filled circle), the $\sum_{\text{s}(\text{C})}{\text{AOSP}}_{\text{Rad}}$ of hesperetin fit the regression model well, yielding *R*^2^=0.942, SE=0.059, and SE_cv_=0.073 (after exclusion of quercetin)

More precisely, in my paper (1), I detected hesperetin as an outlier in regression models for the estimation of both oxidation potential (*E*_p1_) and antioxidant activities (AA), on a set of 14 flavonoids. The models [Models 2 and 7, Figures 2 and 3 in (1)] were based on the sum of atomic orbital spin populations over the carbon atoms in the skeleton of a flavonoid radical, $\sum_{\text{s}(\text{C})}{\text{AOSP}}_{\text{Rad}}$ , calculated using semiepirical PM6 method. Later, in our paper on *E*_p1_ models for 29 flavonoids (2), we succeeded in resolving a problem with hesperetin and its glycosides, hesperidin and neohesperidin, thanks to studies on the electron donation potential of the *ortho*-methoxy group in quinones (3, 4). When we fixed the methoxy group, placing it outside of the plane (orthogonally to the B ring) during optimization, the calculated $\sum_{\text{s}(\text{c})}{\text{AOSP}}_{\text{Rad}}$ for hesperetin, hesperidin, and neohesperidin fit into the model perfectly, Figure 1 in (2) [see more details about approaching certain flavonoids, like flavanones, isoflavones, and flavonoids with *O*-glycosyl, galloyl and methoxy substituents, as well as a new models that we introduced in (2, 5, 6)].

Figures 1 and 2 show that $\sum_{\text{s}(\text{C})}{\text{AOSP}}_{\text{Rad}}$ values for hesperetin calculated in this way fit the quadratic regression models in (1).
